# Attitudes of nursing students toward male and LGBTQ individuals entering the midwifery profession and their role in providing midwifery care

**DOI:** 10.20407/fmj.2025-035

**Published:** 2026-09-25

**Authors:** Mikiko Shimizu, Keisuke Iwase, Hironori Tsuzuki

**Affiliations:** 1 Faculty of Nursing, Fujita Health University, School of Health Sciences, Toyoake, Aichi, Japan; 2 Department of Nursing, Toyohashi Sozo University, Toyohashi, Aichi, Japan

**Keywords:** Gender identity, Sex-based restriction, Male midwife, Midwifery care, Nursing students

## Abstract

**Objective:**

The objective of this study, which targeted nursing students, was to evaluate their level of agreeableness toward males and LGBTQ individuals in the provision of midwifery care.

**Methods:**

A survey request was sent to 562 nursing students at a Private University A, and 99 valid responses (male: 10; female: 89) were received. Due to the small number of male respondents, the analysis was limited to female respondents. An online survey was conducted using Google Forms between April and May 2024. The survey evaluated participants’ level of agreeableness across 14 items defined by the Japan Midwives Association, including the roles and responsibilities of midwives in midwifery care and the acquisition of midwifery qualifications. A four-point Likert scale ranging from “very disagreeable” to “very agreeable” was used. Analyses were conducted to compare responses across survey items and academic year.

**Results:**

The five items for which 50% or more respondents selected “very disagreeable” or “rather disagreeable” were pregnancy, delivery, postpartum, and menstrual disorder care as well as acquisition of midwifery qualifications. Additionally, first-year students tended to evaluate individuals with a non-female appearance as more disagreeable than students in other academic years.

**Conclusions:**

Female nursing students preferred individuals with a female appearance to provide pregnancy, delivery, postpartum and menstrual disorder care and to acquire midwifery qualifications. Moreover, first-year students tended to exhibit higher levels of disagreeableness than students in other academic years.

## Introduction

Midwives in Japan used to be called “*sanba*” (literally, “older woman who assists childbirth”) and were responsible for caring for women during delivery and the postpartum period, as well as for assisting with newborn care. Male *sanba* existed throughout the Edo period and until around the mid-Meiji era.^1^ However, the profession effectively became female-only when the “Regulations for Midwives, Examination Regulations for Midwives, and Registration Regulations for the Midwife Register,” enacted in 1899, stipulated that *sanba* be “females aged 20 years or older.” Later, the term “*sanba”* was changed to “*josanpu*” (literally, “woman who assists childbirth”). In 2002, Article 3 of the Act on Public Health Nurses, Midwives, and Nurses further revised the term to “*josanshi*” (literally, “person who assists childbirth”). Although this change prompted debate regarding male midwives, no legal revisions were made to alter the female-exclusive nature of the profession. In 2005, the Japanese Midwives Association expressed conditional support for the introduction of male midwives, including legal arrangements guaranteeing the right to choose whether one’s midwife is female or male.^2^ Despite more than 20 years having passed since this position was articulated, only females can currently become midwives in Japan.

In recent years, there has been increased global debate and legal developments regarding sexual diversity, including lesbian, gay, bisexual, transgender, and questioning (LGBTQ) individuals. Sexual minorities are estimated to account for approximately 9.7% of the population in Japan.^3^ In 2023, the Japanese Supreme Court ruled that the “Act on Special Cases in Handling Gender Status for Persons with Gender Identity Disorder,” which required individuals seeking legal gender change to have no reproductive glands or to have glands with permanently removed function, was unconstitutional.^4^ This ruling highlights the urgent need to establish frameworks that accommodate sexual diversity. Given that approximately 10% of the population comprises sexual minorities, distinctions cannot be made solely on the basis of “male” or “female.” For example, If an individual assigned female at birth qualifies as a midwife but later undergoes gender transition and is legally registered as male, that individual may lose midwifery qualification under the current legal system. This situation raises concerns about being compelled to continue working under a female designation despite internal gender identity conflict. In contrast, many countries, such as the US and the UK, allow individuals to obtain midwifery qualifications regardless of gender identity or sexual orientation. As a result, such individuals can be active in the clinical field without sex-based restrictions.^5^

As discussions on sexual diversity continue, the current situation in Japan—whereby only females can legally become midwives—requires re-evaluation, particularly given that more than 20 years have passed since the Japanese Midwives Association stated its opinion regarding the introduction of male midwives and that sexual minorities now constitute approximately 10% of the population. Therefore, the objective of this study, which targeted nursing students, was to evaluate the level of agreeableness toward males and LGBTQ individuals in the provision of midwifery care in Japan. Nursing students were selected because they are likely to be involved in midwifery care in the future, either as midwives or nurses in obstetrics settings. They may also provide perinatal care and potentially become recipients of midwifery care themselves. Additionally, this study analyzed responses by academic year (first through fourth year) to examine changes in perceptions based on the progression of their educational program. These results may inform the timing and content of effective educational interventions to promote understanding of sexual diversity in midwife and basic nursing education.

This study examined how nursing students, as potential future providers or recipients of midwifery care, evaluate the agreeableness of males or LGBTQ individuals providing midwifery care in Japan. The findings are expected to contribute to discussions on midwife education, the introduction of male midwives, and the development of a midwifery system that incorporates sexual diversity while also ensuring the right of choice for women receiving care.

## Research methods

### Research design

Cross-sectional study

### Subjects

First- to fourth-year university students of the Faculty of Nursing of Private University A who provided consent to participate in this study.

### Survey period

April through May 2024

### Survey methods

An anonymous online survey was conducted using Google Forms. Survey invitations were distributed via university email and in-person explanations. The questionnaire used in this study was developed by the principal investigator based on materials published by the Japanese Midwives Association regarding the roles and responsibilities of midwives. Because first-year students had not yet studied maternal nursing, selected items were given very simple and non-technical explanations to minimize misunderstandings of specialized terminology. These explanations were limited to minimal background information for accurate interpretation of the response options. The explanations did not provide specialized knowledge or details regarding clinical judgments. All content was standardized to a level appropriate for comprehension by the general population. To secure content validity, a faculty member specializing in maternal nursing reviewed each questionnaire item for accuracy, clarity, and comprehensibility for novice learners. The research team revised the questionnaire based on feedback, and consensus was reached regarding the appropriateness of the expressions used and content of each item.

### Survey items

Participants were asked to respond regarding the level of agreeableness toward individuals with nine gender identities and sexual orientations, including heterosexuals performing the 14 roles and responsibilities of midwives as defined by the Japanese Midwives Association^6^ (Supplementary Material 1), as well as obtaining midwife qualifications. Responses were recorded using a four-point Likert scale ranging from “1=very disagreeable” to “4=very agreeable.” Higher scores indicated greater agreeableness with a midwife of that gender identity performing midwifery care or obtaining midwife qualifications.

There was a possibility that respondents could become both “providers of care” and “recipients of care” in the future. Therefore, they were allowed to respond from either perspective when completing the survey form. They were instructed to evaluate agreeableness based on their current perceptions while considering both possibilities.

The following terms were used in this study for the attributes of the nine gender identities and sexual orientations.

• Female (heterosexual: female)
• Male (heterosexual: male)
• Female (homosexual: lesbian)
• Male (homosexual: gay)
• Female (bisexual: bisexual female)
• Male (bisexual: bisexual male)
• Transgender female (assigned male at birth; identifies as female: transgender female)
• Transgender male (assigned female at birth; identifies as male: transgender male)
• Questioning (individuals whose gender identity or sexual orientation is not yet decided: questioning)

The respondents were asked about attributes comprising sex, age, and university year.

### Analysis methods

Statistical analyses were performed using IBM SPSS Statistics (version 27). Cross-tabulations were performed for each item. Analysis by university year was performed only for items with over 50% of responses indicating “1=very disagreeable” or “2=rather disagreeable.” For the analysis, participants were divided into a low agreeableness group (“1=very disagreeable” and “2=rather disagreeable”) and a high agreeableness group (“3=agreeable” and “4=very agreeable”). Cross-tabulations and Fisher’s exact test were performed to examine differences in responses across academic years between these groups. Adjusted residuals were calculated for items that showed a significant difference on these analyses. All tests were two-tailed, and statistical significance was set at p<5.

### Ethical considerations

This study was conducted with approval from the president of Fujita Health University based on a review by the Fujita Health University Medical Research Ethics Committee (Approval No. HM24-270). In this study, careful considerations were given to the potential psychological impact of terminology related to gender identity on affected individuals. Therefore, the terms “reject” or “accept” were not used. Instead, the terms “agreeable” and “disagreeable” were used in a unified manner in accordance with the responses on the questionnaire.

## Results

### Respondent background

A survey request was sent to 562 nursing students, and 99 valid responses (males: 10; females: 89) were received (valid response rate: 17.6%). Because the number of male respondents was substantially smaller than that of females, the analysis was restricted to female respondents to ensure statistical power and avoid estimation instability. The mean age of participants was 20.0±1.2 years (mean±SD). By academic year, the number of subjects was as follows: first-year students: 20 subjects; second-year students: 24 subjects; third-year students: 24 subjects; fourth-year students: 21 subjects.

### Care practices for which “1. Very disagreeable” or “2. Rather disagreeable” accounted for 50% or more of responses (Figure 1–4)

Of the 14 items, the four care practices for which “1. Very disagreeable” or “2. Rather disagreeable” accounted for 50% or more of responses were pregnancy care, delivery care, postpartum care, and menstrual disorder care.

For pregnancy care, “1=very disagreeable” or “2=rather disagreeable” accounted for 50% or more of responses regarding males, gay males, bisexual males, and questioning people. For delivery care, “1=very disagreeable” or “2=rather disagreeable” accounted for 50% or more of responses regarding males, gay males, bisexual males, transgender females, transgender males, and questioning people. For postpartum care, “1=very disagreeable” or “2=rather disagreeable” accounted for 50% or more of responses regarding males, gay males, bisexual males, transgender females, transgender males, and questioning people. For menstrual disorder care, “1=very disagreeable” or “2=rather disagreeable” accounted for 50% or more of responses regarding males, gay males, and bisexual males.

### Care practices for which “1. Very disagreeable” or “2. Rather disagreeable” accounted for 50% or more of responses for agreeableness regarding acquisition of midwifery qualifications (Figure 5)

For evaluation of agreeableness regarding acquiring midwifery qualifications, “1. Very disagreeable” or “2. Rather disagreeable” accounted for 50% or more of responses regarding males and bisexual males.

### Evaluation of the agreeableness regarding midwifery care and acquiring midwifery qualifications by university year (Table 1)

For pregnancy care, significant differences were observed in response distribution by university year in the low agreeableness group and the high agreeableness group for males, lesbians, gay males, bisexual females, transgender females, transgender males, bisexual males, and questioning people. In the low agreeableness group, responses regarding lesbians and bisexual males were significantly higher than the expected frequency for first-year students (adjusted residuals: 2.7, 2.7), whereas responses regarding males, gay males, transgender females, and transgender males were significantly lower than the expected frequency for second-year students (adjusted residuals: –3.0, –3.3, –3.3, –3.2). Moreover, responses regarding bisexual males and questioning people were significantly higher than the expected frequency for first-year students (adjusted residuals: 2.0, 3.0) but significantly lower than the expected frequency for second-year students (adjusted residuals: –3.2, –2.5).

For delivery care, significant differences were observed in response distribution by university year in the low agreeableness group and the high agreeableness group for bisexual females and questioning people. In the low agreeableness group, responses regarding questioning people were significantly higher than the expected frequency for first-year students (adjusted residual: 3.0). Moreover, responses regarding bisexual females were significantly higher than the expected frequency for first-year students (adjusted residual: 2.8) but significantly lower than the expected frequency for second-year students (adjusted residual: –2.3).

For postpartum care, significant differences were observed in response distribution by university year in the low agreeableness group and the high agreeableness group for bisexual females. In the low agreeableness group, responses were significantly higher than the expected frequency for first-year students (adjusted residual: 2.8).

For menstrual disorder care, significant differences were observed in response distribution by university year in the low agreeableness group and the high agreeableness group for lesbians and bisexual females. In the low agreeableness group, responses regarding lesbians were significantly higher than the expected frequency for first-year students (adjusted residual: 2.5). Moreover, responses regarding bisexual females were significantly higher than the expected frequency for first-year students (adjusted residual: 2.2) but significantly lower than the expected frequency for second-year students (adjusted residual: –2.0).

For agreeableness regarding acquiring midwifery qualifications, significant differences were observed in response distribution by university year in the low agreeableness group and the high agreeableness group for gay males. In the low agreeableness group, responses were significantly lower than the expected frequency for second-year students (adjusted residual: –2.1) and significantly higher than the expected frequency for fourth-year students (adjusted residual: 2.1).

## Discussion

The novelty of this study lies in the fact that it targeted nursing students to investigate the evaluation of the agreeableness of males and LGBTQ individuals providing midwifery care in Japan.

### Evaluation of agreeableness regarding midwifery care (14 Items)

For pregnancy, delivery, postpartum care, and menstrual disorder care, “1=very disagreeable” or “2=rather disagreeable” accounted for 50% or more of responses regarding males, gay males, and bisexual males. These findings indicate that individuals with a female appearance were more likely to be perceived as agreeable providers for such types of care. However, although transgender females and transgender males may physically appear to be female, responses indicated a tendency toward disagreeableness for these groups. Furthermore, the fact that questioning individuals also tended to be considered as disagreeable indicated a negative tendency not only towards individuals with a male appearance but also individuals with a female or ambiguous appearance. Suzuki (2017)^7^ reported that individuals belonging to the same gender category are often perceived as having a shared sense of values or anticipated to have such values. Previous studies have also shown that women may experience embarrassment and discomfort when receiving care from male midwives.^8–10^ These findings suggest that care provided by individuals perceived as being of the same sex may be considered more agreeable, as this perception may reduce feelings of embarrassment and discomfort associated with interactions with the opposite sex. Therefore, the female recipients of such care need to be given consideration, such as providing them with the right to choose a male or female midwife.

For evaluation of the ten items other than pregnancy, delivery, postpartum care, and menstrual disorder care. However, over 50% of responses indicated “agreeable” or “very agreeable.” An international study reported that one reason for preferring male midwives is the fact that male midwives are kinder than female midwives.^11^ Thus, midwifery care that was evaluated as being agreeable could be provided to female recipients in the same manner as a female midwife provided.

### Evaluation of agreeableness regarding midwifery care by university year

Evaluation of agreeableness regarding midwifery care by academic year indicated that first-year students had the highest response rate for disagreeableness compared to students in other academic years. In contrast, second-year students showed the highest response rate for agreeableness compared with other students in other academic years, as a tendency was observed for disagreeable evaluation to again rise in third-year and fourth-year students. First-year students had not yet completed any coursework and therefore, had limited opportunities to acquire knowledge and skills regarding the perinatal period. Moreover, because data were collected 1–2 months after university enrollment, first-year students were very likely highly interested in healthcare professionals but had minimal general nursing knowledge or skills. Second-year students had acquired classroom knowledge in maternal nursing because they had completed coursework in introductory maternal nursing during the first semester and maternal nursing theories during the second semester. The introductory maternal nursing course includes lectures on gender, in which students learn about topics including sexual diversity and sex education. The second-year students targeted in the present study had already attended over half of the lectures regarding “Concepts of Sexual and Reproductive Health and Sexuality” in the introductory maternal nursing. Third-year students had earned credits on maternal nursing skills in the first semester, gaining skills as well as knowledge regarding maternal nursing. In the second semester, third-year students sequentially engaged in hospital practice for two weeks for coursework on maternal nursing practice. This gave them opportunities to observe postpartum women and newborns in obstetric wards and provide nursing support. Many students also engaged with pregnant women during outpatient visits and were present during deliveries. All fourth-year students had completed the coursework required for first year through third year. Based on such an education curriculum, it is assumed that first-year students’ responses were based primarily on general knowledge of the perinatal period. In contrast, second-year students provided responses based on more specialized knowledge regarding sex. As Tian et al. (2022)^12^ and Gerezgihe et al. (2022)^13^ reported that health education improves acceptance of cervical cancer checkups and medical service attendance, the acquisition of knowledge related to sex among second-year students may have resulted in higher levels of agreeable evaluation. However, as they had not yet completed all maternal nursing coursework, it is difficult to fully evaluate their level of knowledge. Third-year students received skills training, and fourth-year students had opportunities to participate in clinical midwifery practice during ward rotations. Therefore, they were likely able to develop a more concrete understanding of midwifery care compared with the abstract knowledge gained by second-year students. Such an image may have been reflected in their responses. In recent years, male midwives are contributing to perinatal care, and the percentage of male midwives has increased amongst perinatal healthcare providers worldwide.^11^ Given this global trend, it is important to identify factors—such as lecture content, experiences during clinical practice, and instructor involvement—that influence changes in agreeableness evaluations across academic years. Therefore, these findings should be used to inform educational interventions aimed at addressing factors that contribute to decreased agreeableness, especially after clinical practice, and to improve educational programs that encourage students to embrace sexual diversity and midwifery care concepts in a positive manner. Further investigations also need to be conducted to enhance perceptions of the agreeableness not only among males but also among LGBTQ people providing midwifery in Japan.

### Evaluation of agreeableness regarding acquiring midwifery qualifications

The results for evaluation of agreeableness regarding acquiring midwifery qualifications suggested that, similar to midwifery care, disagreeableness was high towards males and bisexual males, indicating that there was a strong disagreeable evaluation of midwives who had a male appearance. When analyzing academic year, the highest level of disagreeableness regarding gay males was observed among fourth-year students compared with students of other years. This was the only item for which evaluation was more disagreeable than for first-year students. to the fact that gay males rather than males were being targeted and received more disagreeable evaluations among fourth-year students may suggest that they might have experienced something unexpected during their hospital practice or been influenced by some personal experience in their private life. However, as the underlying reasons for this observation remain unclear, future research is needed to discuss these topics.

### Study limitations

This study has the following three limitations. First, although differences in agreeableness evaluation by university year were observed, changes to agreeableness evaluation of individual nursing students were not able to be clarified due to the cross-sectional nature of this study. Because participants differed across academic years, comparisons between groups cannot be interpreted as longitudinal changes. Future longitudinal research needs to be conducted to clarify individual changes in evaluation. Second, in the evaluation of agreeableness regarding acquiring midwifery qualifications, the result that fourth-year students exhibited the highest level of disagreeableness toward gay males cannot be fully explained. The factors that influence such results need to be clarified in the future. Third, the relatively small sample size may have increased the influence of individual variability on the results. Because the number of male respondents was limited, males were excluded from the analysis to secure statistical power and avoid estimation instability. However, future research should investigate how male nursing students, who may be involved in perinatal nursing care in the future, evaluate agreeableness.

## Conclusions

Female nursing students tended to prefer individuals with a female appearance to provide pregnancy, delivery, postpartum care, and menstrual disorder care and to acquire midwifery qualifications. These items tended to be evaluated more often as disagreeable by first-year students than students in other academic years.

## Figures and Tables

**Figure 1:**
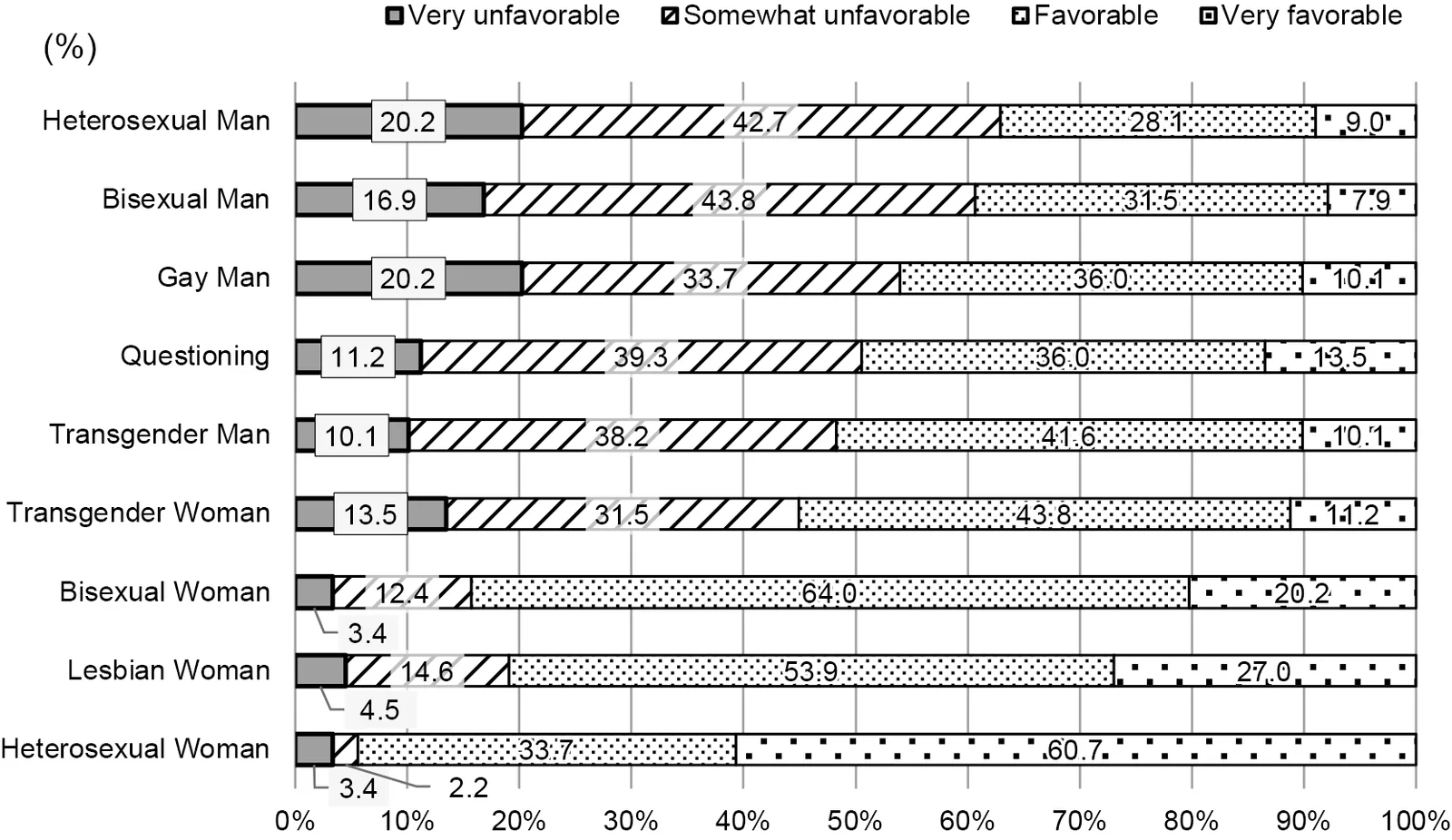
Prenatal care

**Figure 2:**
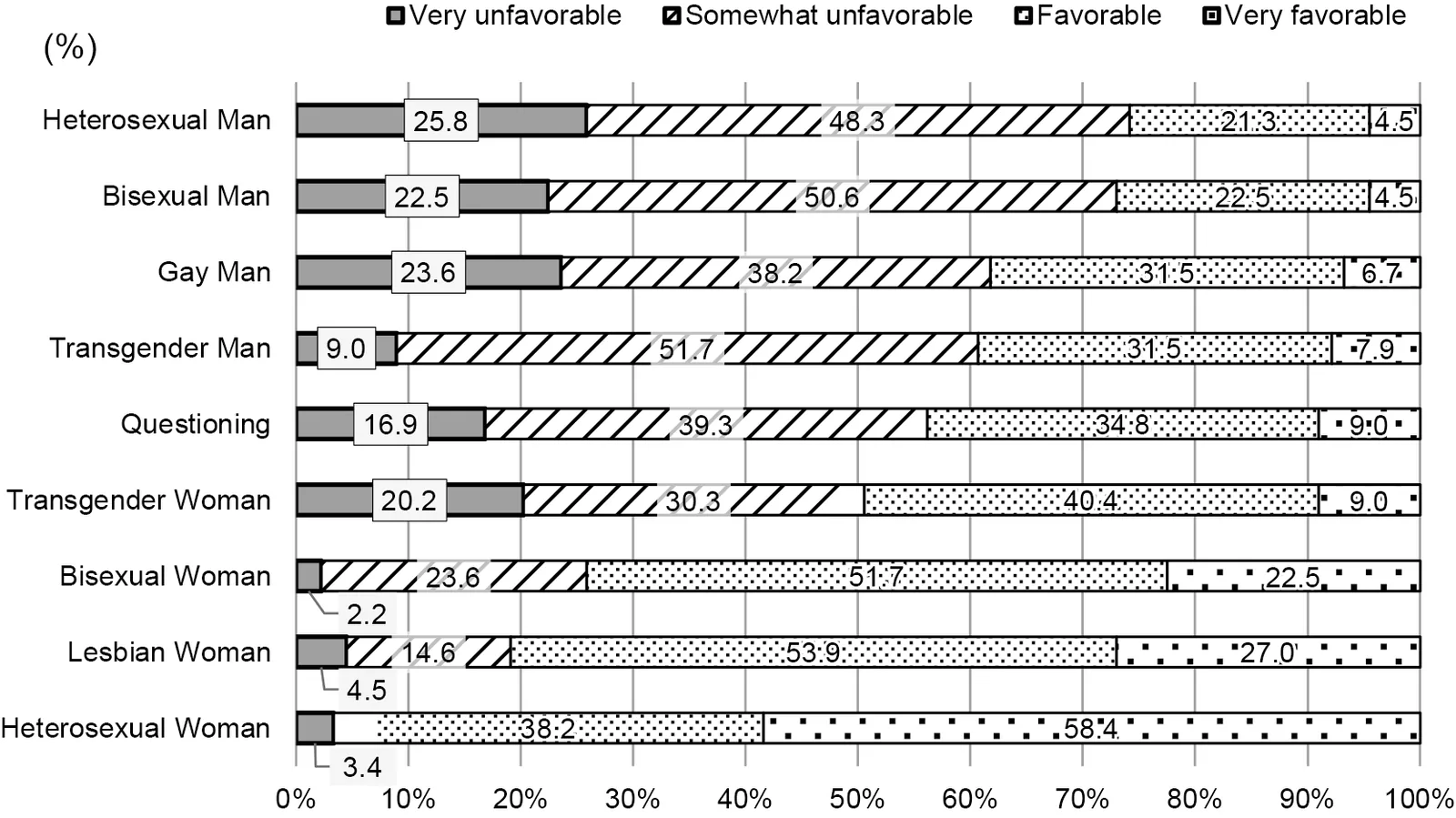
Childbirth care

**Figure 3:**
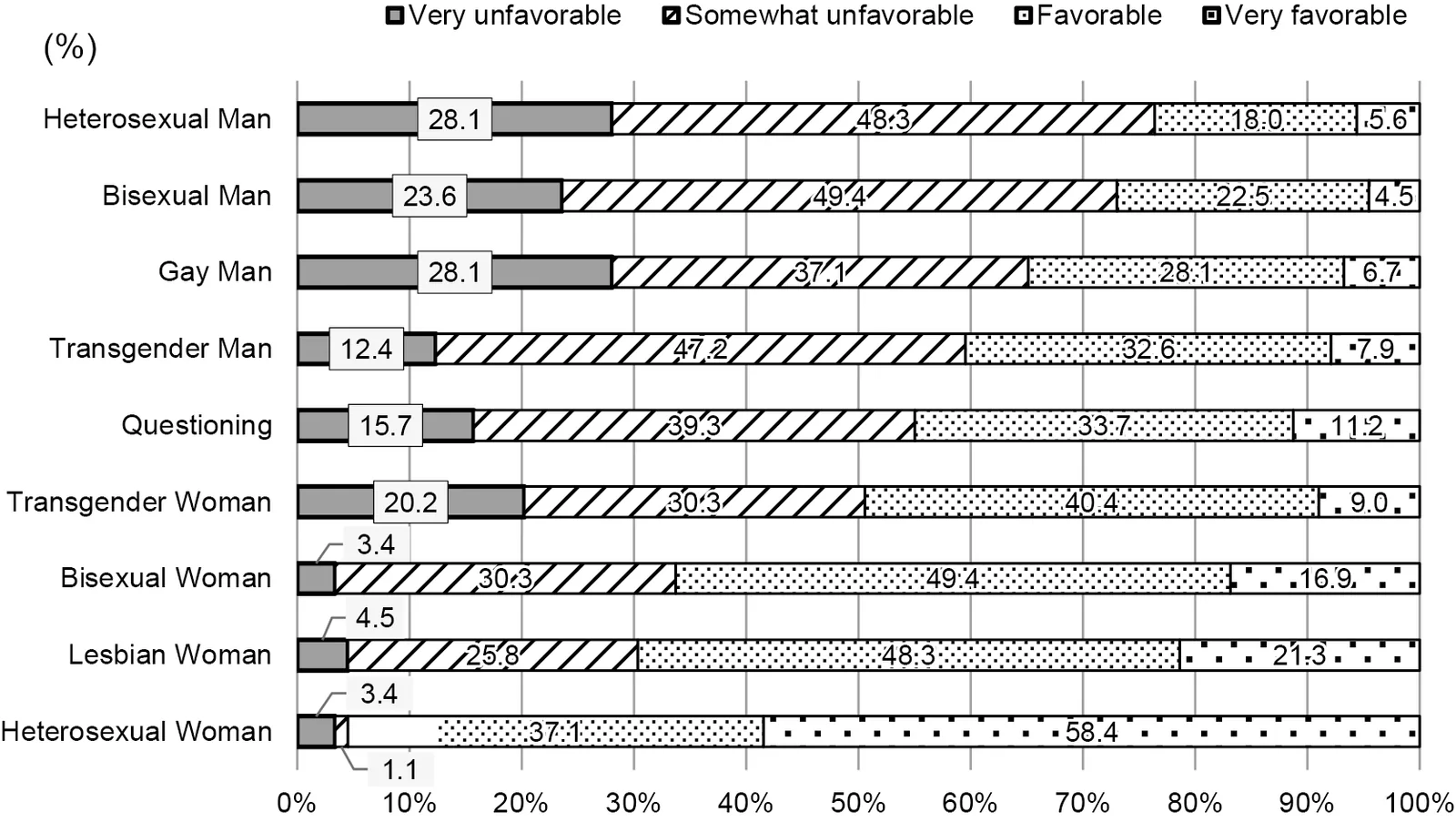
Postpartum care

**Figure 4:**
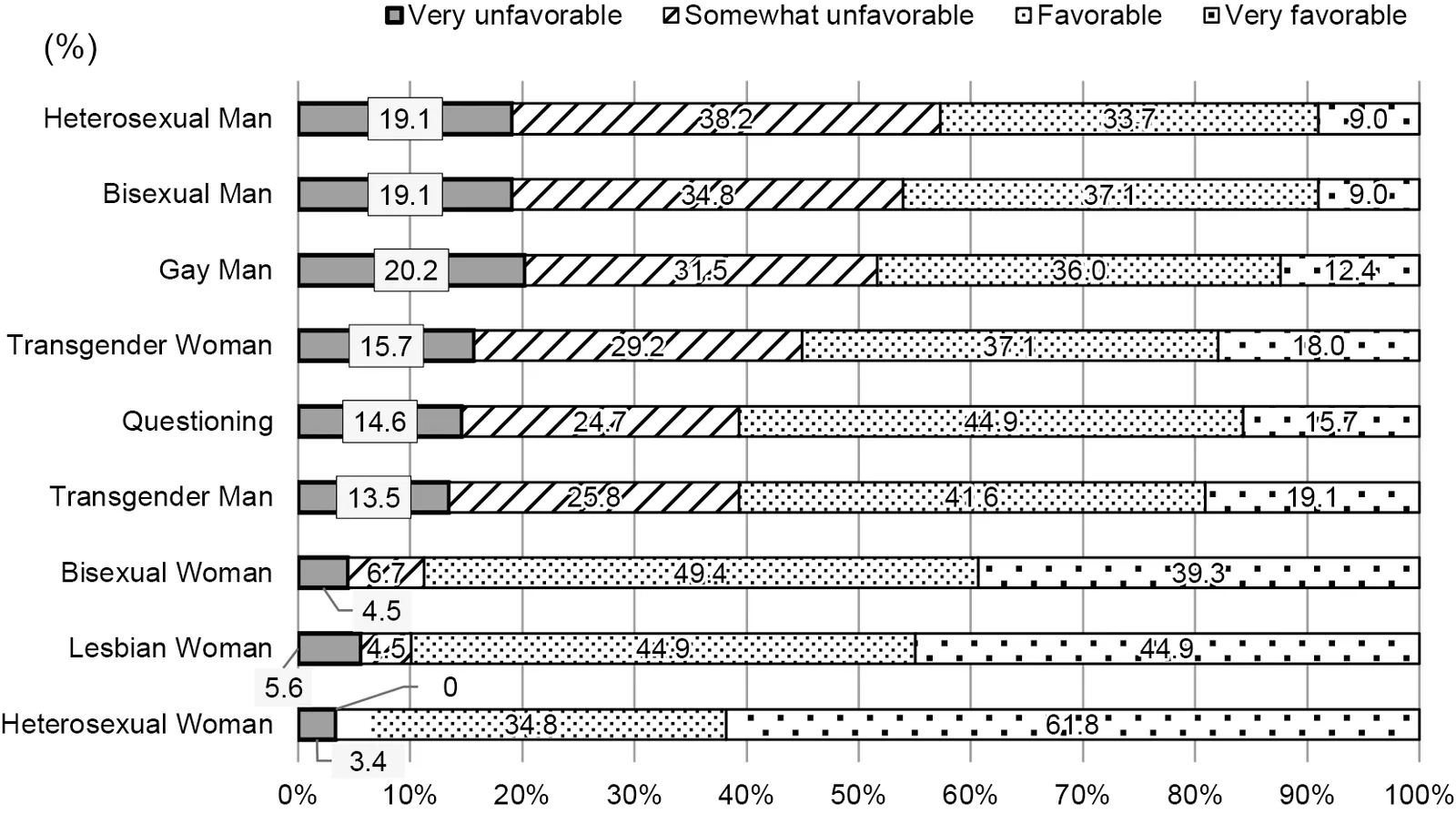
Menstrual disorders care

**Figure 5:**
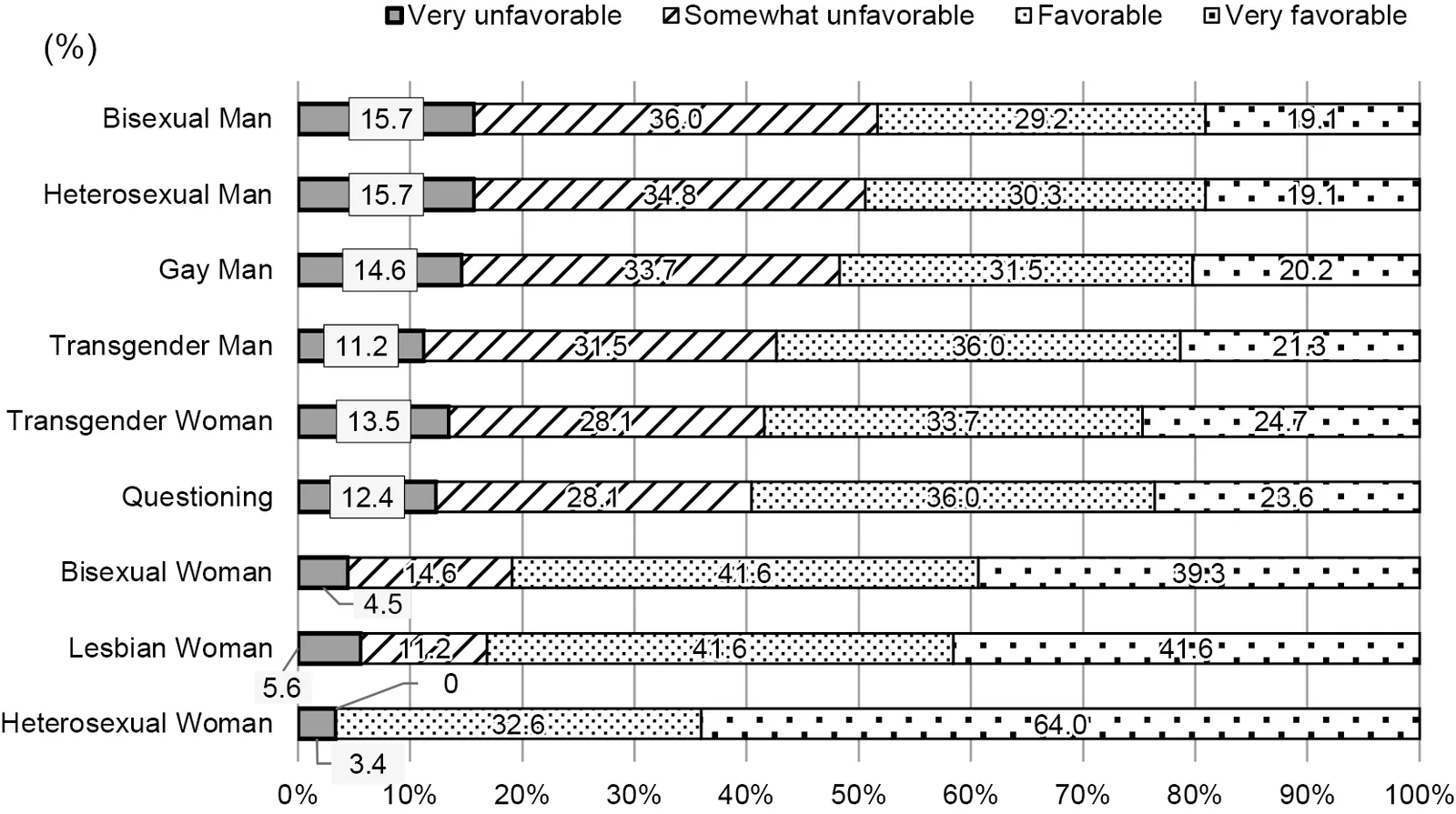
Acquisition of Midwifery Qualification

**Table 1:** Midwifery care and Attitudes Toward Obtaining Midwifery Certification by Grade Level

			Grade1(N=20)		Grade2(N=24)		Grade3(N=24)		Grade4(N=21)		χ^2^ test
			N	%	N	%	N	%	N	%	
Prenatal care	Heterosexual Woman	Negative*	1	5.0%	1	4.2%	1	4.2%	2	9.5%	
		Positive**	19	95.0%	23	95.8%	23	95.8%	19	90.5%	
	Heterosexual Man	Negative	16	80.0%	9	37.5%	15	62.5%	16	76.2%	p<0.05
		Positive	4	20.0%	15	62.5%	9	37.5%	5	23.8%	
	Lesbian Woman	Negative	8	40.0%	2	8.3%	2	8.3%	5	23.8%	p<0.05
		Positive	12	60.0%	22	91.7%	22	91.7%	16	76.2%	
	Gay Man	Negative	14	70.0%	6	25.0%	14	58.3%	14	66.7%	p<0.01
		Positive	6	30.0%	18	75.0%	10	41.7%	7	33.3%	
	Bisexual Woman	Negative	7	35.0%	1	4.2%	2	8.3%	4	19.0%	p<0.05
		Positive	13	65.0%	23	95.8%	22	91.7%	17	81.0%	
	Bisexual Man	Negative	16	80.0%	8	33.3%	15	62.5%	15	71.4%	p<0.01
		Positive	4	20.0%	16	66.7%	9	37.5%	6	28.6%	
	Transgender Woman	Negative	11	55.0%	4	16.7%	14	58.3%	11	52.4%	p<0.05
		Positive	9	45.0%	20	83.3%	10	41.7%	10	47.6%	
	Transgender Man	Negative	13	65.0%	5	20.8%	12	50.0%	13	61.9%	p<0.05
		Positive	7	35.0%	19	79.2%	12	50.0%	8	38.1%	
	Questioning	Negative	16	80.0%	7	29.2%	11	45.8%	11	52.4%	p<0.01
		Positive	4	20.0%	17	70.8%	13	54.2%	10	47.6%	
Childbirth care	Heterosexual Woman	Negative	1	5.0%	0	0.0%	1	4.2%	1	4.8%	
		Positive	19	95.0%	24	100.0%	23	95.8%	20	95.2%	
	Heterosexual Man	Negative	18	90.0%	15	62.5%	18	75.0%	15	71.4%	
		Positive	2	10.0%	9	37.5%	6	25.0%	6	28.6%	
	Lesbian Woman	Negative	10	50.0%	5	20.8%	5	20.8%	7	33.3%	
		Positive	10	50.0%	19	79.2%	19	79.2%	14	66.7%	
	Gay Man	Negative	16	80.0%	10	41.7%	15	62.5%	14	66.7%	
		Positive	4	20.0%	14	58.3%	9	37.5%	7	33.3%	
	Bisexual Woman	Negative	10	50.0%	2	8.3%	4	16.7%	7	33.3%	p<0.01
		Positive	10	50.0%	22	91.7%	20	83.3%	14	66.7%	
	Bisexual Man	Negative	17	85.0%	14	58.3%	18	75.0%	16	76.2%	
		Positive	3	15.0%	10	41.7%	6	25.0%	5	23.8%	
	Transgender Woman	Negative	12	60.0%	9	37.5%	13	54.2%	11	52.4%	
		Positive	8	40.0%	15	62.5%	11	45.8%	10	47.6%	
	Transgender Man	Negative	16	80.0%	12	50.0%	13	54.2%	13	61.9%	
		Positive	4	20.0%	12	50.0%	11	45.8%	8	38.1%	
	Questioning	Negative	17	85.0%	11	45.8%	11	45.8%	11	52.4%	p<0.05
		Positive	3	15.0%	13	54.2%	13	54.2%	10	47.6%	
Postpartum care	Heterosexual Woman	Negative	1	5.0%	1	4.2%	1	4.2%	1	4.8%	
		Positive	19	95.0%	23	95.8%	23	95.8%	20	95.2%	
	Heterosexual Man	Negative	16	80.0%	18	75.0%	19	79.2%	15	71.4%	
		Positive	4	20.0%	6	25.0%	5	20.8%	6	28.6%	
	Lesbian Woman	Negative	11	55.0%	5	20.8%	5	20.8%	6	28.6%	p<0.05
		Positive	9	45.0%	19	79.2%	19	79.2%	15	71.4%	
	Gay Man	Negative	14	70.0%	13	54.2%	17	70.8%	14	66.7%	
		Positive	6	30.0%	11	45.8%	7	29.2%	7	33.3%	
	Bisexual Woman	Negative	12	60.0%	7	29.2%	5	20.8%	6	28.6%	p<0.05
		Positive	8	40.0%	17	70.8%	19	79.2%	15	71.4%	
	Bisexual Man	Negative	16	80.0%	17	70.8%	18	75.0%	14	66.7%	
		Positive	4	20.0%	7	29.2%	6	25.0%	7	33.3%	
	Transgender Woman	Negative	10	50.0%	9	37.5%	14	58.3%	12	57.1%	
		Positive	10	50.0%	15	62.5%	10	41.7%	9	42.9%	
	Transgender Man	Negative	14	70.0%	13	54.2%	14	58.3%	12	57.1%	
		Positive	6	30.0%	11	45.8%	10	41.7%	9	42.9%	
	Questioning	Negative	15	75.0%	10	41.7%	13	54.2%	11	52.4%	
		Positive	5	25.0%	14	58.3%	11	45.8%	10	47.6%	
Menstrual disorders care	Heterosexual Woman	Negative	1	5.0%	0	0.0%	1	4.2%	1	4.8%	
		Positive	19	95.0%	24	100.0%	23	95.8%	20	95.2%	
	Heterosexual Man	Negative	12	60.0%	11	45.8%	12	50.0%	16	76.2%	
		Positive	8	40.0%	13	54.2%	12	50.0%	5	23.8%	
	Lesbian Woman	Negative	5	25.0%	0	0.0%	1	4.2%	3	14.3%	p<0.05
		Positive	15	75.0%	24	100.0%	23	95.8%	18	85.7%	
	Gay Man	Negative	10	50.0%	9	37.5%	12	50.0%	15	71.4%	
		Positive	10	50.0%	15	62.5%	12	50.0%	6	28.6%	
	Bisexual Woman	Negative	5	25.0%	0	0.0%	1	4.2%	4	19.0%	p<0.05
		Positive	15	75.0%	24	100.0%	23	95.8%	17	81.0%	
	Bisexual Man	Negative	12	60.0%	9	37.5%	12	50.0%	15	71.4%	
		Positive	8	40.0%	15	62.5%	12	50.0%	6	28.6%	
	Transgender Woman	Negative	8	40.0%	8	33.3%	11	45.8%	13	61.9%	
		Positive	12	60.0%	16	66.7%	13	54.2%	8	38.1%	
	Transgender Man	Negative	10	50.0%	7	29.2%	8	33.3%	10	47.6%	
		Positive	10	50.0%	17	70.8%	16	66.7%	11	52.4%	
	Questioning	Negative	10	50.0%	8	33.3%	8	33.3%	9	42.9%	
		Positive	10	50.0%	16	66.7%	16	66.7%	12	57.1%	
Attitudes Toward Obtaining Midwifery Certification	Heterosexual Woman	Negative	1	5.0%	0	0.0%	1	4.2%	1	4.8%	
		Positive	19	95.0%	24	100.0%	23	95.8%	20	95.2%	
	Heterosexual Man	Negative	11	55.0%	8	33.3%	11	45.8%	15	71.4%	
		Positive	9	45.0%	16	66.7%	13	54.2%	6	28.6%	
	Lesbian Woman	Negative	6	30.0%	1	4.2%	5	20.8%	3	14.3%	
		Positive	14	70.0%	23	95.8%	19	79.2%	18	85.7%	
	Gay Man	Negative	10	50.0%	7	29.2%	11	45.8%	15	71.4%	p<0.05
		Positive	10	50.0%	17	70.8%	13	54.2%	6	28.6%	
	Bisexual Woman	Negative	6	30.0%	1	4.2%	4	16.7%	6	28.6%	
		Positive	14	70.0%	23	95.8%	20	83.3%	15	71.4%	
	Bisexual Man	Negative	12	60.0%	8	33.3%	11	45.8%	15	71.4%	
		Positive	8	40.0%	16	66.7%	13	54.2%	6	28.6%	
	Transgender Woman	Negative	9	45.0%	7	29.2%	8	33.3%	13	61.9%	
		Positive	11	55.0%	17	70.8%	16	66.7%	8	38.1%	
	Transgender Man	Negative	11	55.0%	6	25.0%	9	37.5%	12	57.1%	
		Positive	9	45.0%	18	75.0%	15	62.5%	9	42.9%	
	Questioning	Negative	11	55.0%	6	25.0%	8	33.3%	11	52.4%	
		Positive	9	45.0%	18	75.0%	16	66.7%	10	47.6%	

*: Negative:includes ‘Very unfavorable’ or ‘Somewhat unfavorable’ **: Positive:incluges ‘Very favorable’ or ‘Favorable’

## Supplementary Material

PDF-Japanese
