# Supplementary material for: Attitudes of nursing students toward male and LGBTQ individuals entering the midwifery profession and their role in providing midwifery care
Source: Fujita Med J. 2026 Sep 25;12(4):317–24. doi: 10.20407/fmj.2025-035 (PMC13724197; doi:10.20407/fmj.2025-035)
Supplement: Supplementary file 1 [file fmj-12-317_s1.pdf]

タイトル：

看護学生を対象とした男性や LGBTQ の人が助産師になることに対する意識調査
―助産ケアの実施―

Attitudes of Nursing Students toward Men and LGBTQ Individuals Entering the Midwifery Profession and Their Role in Providing Midwifery Care

ランニングタイトル：男性や LGBTQ の人が助産ケアを実施することに対する意識調査
Attitudes of Men and LGBTQ Individuals Providing Midwifery Care

著者：

Mikiko Shimizu, RN, PhD [1], Keisuke Iwase, RN, MS [1], Hironori Tsuzuki, RN, MS [2]

[1] Faculty of Nursing, Fujita Health University, School of Health Sciences, Toyoake, Aichi, Japan
[2] Department of Nursing, Toyohashi Sozo University, Toyohashi, Aichi, Japan

Corresponding author: Mikiko Shimizu, RN, PhD
Faculty of Nursing, Fujita Health University, School of Health Sciences, 1-98,
Dengakugakubo, Kutsukake-cho, Toyoake, Aichi 470-1192, Japan
E-mail: mshimizu@fujita-hu.ac.jp

要約

【目的】

本研究は、看護学生を対象として男性および LGBTQ にある人が助産ケアを実施することに対する意識を明らかにすることを目的とした。

【研究方法】

私立 A 大学看護学科 1 〜4 年生の女子学生 89 名を対象にして、2024 年 4〜5 月に Google フォームによる Web 調査を実施した。調査項目は日本助産師会が定める助産師の役割・責務の 14 項目の助産ケアおよび助産師資格取得に関する意識であり、リッカートの 4 件法「非常に好ましくない」から「非常に好ましい」を用いた。

【結果】

「非常に好ましくない」「あまり好ましくない」の回答 50%以上が含まれた項目は妊娠/分娩/産褥/月経障害ケアおよび助産師資格取得に関する意識の 5 項目であった。これらの項目を学年別で比較した結果、外見が女性以外において「非常に好ましくない」「あまり好ましくない」の割合が他の学年に比較して 1 年生は高い傾向があった。

【結論】

看護学生は、妊娠/分娩/産褥期ケア、月経障害のケアおよび助産師資格取得において、外見が女性であることを好む傾向があった。学年別では 1 年生が他の学年と比較して否定的な意識が高い傾向があった。

キーワード

性自認（Gender identity）

性別による制限（Sex-based restriction）

男性助産師（ale midwife）

助産ケア（Midwifery Care）

看護学生（Nursing students）

本文

はじめに

日本の助産師はかつて産婆と呼ばれ、分娩期・産褥期の女性や新生児の援助を担っていた。江戸時代・明治時代中期頃までは男性産婆が存在していた[1]。しかし、1899 年に公布された「産婆規則と産婆試験規則、ならびに産婆名簿登録規則」にて、産婆は「満 20 歳以上の女性」と規定されたため、事実上女性独占の職業となった。その後、産婆から助産婦への名称変更を経て 2002 年には保健師助産師看護師法第 3 条によって、助産婦から助産師の名称変更が行われた。この際、男性助産師に関する議論がされたが、女性独占の職業であることは法律上変わらなかった。日本助産師会は、2005 年に妊産婦が女性、男性の助産師を選べる権利を保証する法的整備などの条件付きで男性助産師導入に賛成の見解を示した[2]。この見解より、20 年が経過しているが、女性のみが助産師になれるという現状は変わっていない。

近年、LGBTQ（Lesbian, Gay, Bisexual, Transgender, Questioning）を含む多様な性の在り方に関する議論や法整備は世界的に進展している。日本でも性的マイノリティは人口の約 9.7%を占めると報告されている[3]。2023 年に性別変更において、性同一性障害者の性別の取り扱いの特例に関する法律にて定める性別の取り扱いの変更の要件のうち、「生殖腺がないこと又は生殖腺の機能を永続的に欠く状態にあること」があるのは違憲であるという最高裁の判断が示され[4]、多様な性を前提とした制度整備が急務となっている。これらより、人口の約 1 割が性的マイノリティであり、さらに男女の性別では判断できない現状がある。また、女性のときに助産師免許を取得し、その後性転換をして戸籍上男性になった場合、現行の法律では助産師免許を剥奪される可能性があるため、性転換への葛藤を抱えながら助産師として働かなければならないという問題も生じることが考えられる。一方、アメリカやイギリスなど多くの国では、性自認や性的指向にかかわらず助産師資格を取得できる体制が整えられ、性に縛られることなく臨床現場で活躍されている[5]。

以上のように、20 年以上に渡りほぼ変化が無かったことや性的マイノリティの人口が 1 割を占めることを踏まえて、法律上女子のみが助産師になることができる今日の日本の現状を検討する必要があると考える。そこで、本研究では看護学生を対象として男性および LGBTQ にある人が日本で助産ケアを実施することに対する意識を明らかにすることを目的とした。看護学生を対象とした理由は、近い将来に助産師または看護師となって産科病棟で働き、周産期の援助を実施する可能性があることや自分自身が助産ケアの受け手になる可能性があることから助産ケアに関係する人材であると考えたからである。本研究によって、助産ケアの提供または受領側となる可能性のある看護学生の男性および LGBTQ にある人が日本で助産ケアを実施することに対する意識が明らかとなる。これより、近い未来に助産ケア現場に関わる人材の意識を反映させることができる点に本研究の意義があると考える。

研究方法

研究デザイン：横断研究

対象：研究の同意が得られた私立 A 大学看護学科 1～4 年生の大学生とした。

調査期間：2024 年 4～5 月

調査方法

Google フォームを用いた無記名式オンラインアンケート調査を実施し、調査協力は学内メールおよび対面での説明を通じて依頼した。

調査項目

異性愛者を含む 9 つの性自認および性的指向にある人が、日本助産師会[6]で定められている助産師の役割・責務の 14 項目（Supplementary Material 1）の助産ケアを実施することおよび助産師資格を取得することに関する意識について、リッカートの 4 件法で「1.非常に好ましくない」から「4.非常に好ましい」で回答を求めた。数字が高いほど、その性の助産師の助産ケア実施および助産師資格の取得を肯定的に意識していることを意味した。

将来的に「ケアを提供する側」と「ケアの受け手側」の両方になる可能性がある対象となる。調査票ではどちらの立場で回答されるのか限定しておらず、両方になる可能性を踏まえて、回答時のありのままの意識での回答を求めた。

9 つの性自認および性的指向の属性、本研究の使用名は以下の通りである。

・女性（異性愛：女性）

・男性（異性愛：男性）

・女性（同性愛：レズビアン）

・男性（同性愛：ゲイ）

・女性（バイセクシャル：バイセクシャル女性）

・男性（バイセクシャル：バイセクシャル男性）

・トランス女性（身体の性は男性、性自認は女性：トランス女性）

・トランス男性（身体の性は女性、性自認は男性：トランス男性）

・クエスチョニング（自身の性自認や性的指向が定まっていない：クエスチョニング）

回答者の属性として性別、年齢、学年を尋ねた。

分析方法

分析ソフトは SPSS Statistics（version 27）を用いた。各項目についてクロス集計を行った。学年別の分析では「1.非常に好ましくない」および「2.あまり好ましくない」を否定群、「3.好ましい」および「4.非常に好ましい」を肯定群として 2 群に分けて、クロス集計および person の χ^2 検定を行った。

倫理的配慮

本研究は、藤田医科大学医学研究倫理委員会の審査にて学長の承認を得てから実施した（HM24-270）。

結果

回答者の背景

看護学科の学生 562 名に調査を依頼し、99 名（男性 10 名、女性 89 名）から有効回答を得た

（有効回答率 17.6%）。男性回答者が女性に比べて非常に少なかったため、統計的検出力の確保と推定の不安定性を避ける目的で、分析は女性回答者に限定した。年齢は 20.0±1.2(平均値±SD)歳であった。学年別の人数は、1 年生 20 名、2 年生 24 名、3 年生 24 名、4 年生 21 名であった。

「1.非常に好ましくない」または「2.あまり好ましくない」の回答が 50%以上となったケア（Figure 1-4）

「1.非常に好ましくない」または「2.あまり好ましくない」の回答が 50%以上となったケアは、14 項目のケアのうち、妊娠期ケア、分娩期ケア、産褥期ケア、月経障害ケアの 4 項目であった。

妊娠期ケアにおいて、「1.非常に好ましくない」または「2.あまり好ましくない」の回答が 50%以上の属性は、男性、ゲイ、バイセクシャル男性、クエスチョニングであった。分娩期ケアにおいて、「1.非常に好ましくない」または「2.あまり好ましくない」の回答が 50%以上の属性は、男性、ゲイ、バイセクシャル男性、トランス女性、トランス男性、クエスチョニングであった。産褥期ケアにおいて、「1.非常に好ましくない」または「2.あまり好ましくない」の回答が 50%以上の属性は、男性、ゲイ、バイセクシャル男性、トランス女性、トランス男性、クエスチョニングであった。月経障害ケアにおいて、「1.非常に好ましくない」または「2.あまり好ましくない」の回答が 50%以上の属性は、男性、ゲイ、バイセクシャル男性であった。

助産師資格取得に関する意識について「1.非常に好ましくない」または「2.あまり好ましくない」の回答が 50%以上となったケア（Figure 5）

助産師資格取得に関する意識において、「1.非常に好ましくない」または「2.あまり好ましくない」の回答が 50%以上の属性は、男性、バイセクシャル男性であった。

学年別の助産ケアおよび助産師資格取得に関する意識（Table1）

「1.非常に好ましくない」または「2.あまり好ましくない」の回答が 50%以上となったケア 4 項目および助産師資格取得に関する意識 1 項目の合計 5 項目について、学年別にクロス集計および person の χ^2 検定を行った。

妊娠期ケアにおいて、男性、レズ、ゲイ、バイセクシャル女性、バイセクシャル男性、トランス女性、トランス男性、クエスチョニングにおいて有意差があった（$p<0.05$）。分娩期ケアにおいて、バイセクシャル女性、クエスチョニングにおいて有意差があった（$p<0.05$）。産褥期ケアにおいて、レズ、バイセクシャル女性において有意差があった（$p<0.05$）。月経障害ケアにおいて、レズ、バイセクシャル女性において有意差があった（$p<0.05$）。助産師資格取得に関する意識において、ゲイにおいて有意差があった（$p<0.05$）。これらの項目において、妊娠期ケアのトランス女性、助産師資格取得に関する意識のゲイ以外は全て他の学年に比較して、1 年生の否定群の割合が最も高かった。一方、産褥期ケアのバイセクシャル女性以外は全て他の学年に比較して、2 年生の否定群の割合が最も低かった。

考察

本研究の新規性は、看護学生を対象として、男性および LGBTQ にある人が日本で助産ケアを実施することに対する意識調査した点である。

助産ケア（14 項目）の意識について

妊娠／分娩／産褥期ケアおよび月経障害ケアでは、男性、ゲイ、バイセクシャル男性が好まれない結果より、これらのケアにおいては、外見が女性である人が好まれる傾向があった。しかし、トランス女性またはトランス男性も外見が女性である人の可能性があるが、これらの性は否定的な傾向があった。さらに、クエスチョニングも否定的な傾向があったことより、外見が男性である人だけでなく、外見が女性であるか不明瞭な性に関しても否定的な傾向がみられた。鈴木（2017）[7]が同一のジェンダー・カテゴリーに属する人は共通の価値観をもっているかのように知覚されやすく，またもっていることを期待されると述べている。さらに、先行研究では、男性助産師によるケアに対して、女性が羞恥心や不安を感じることが明らかになっている[8-10]。これらより、同性と認識され、共通の価値観をもっていると知覚されて、異性に対する羞恥心や不安がない点で、これらのケアは外見が女性であることが好まれた可能性が示唆された。そのため、これらのケアに関しては、受け手の女性が男性または女性助産師を選択できる権利を提供することなどの配慮が必要になってくると考える。

一方、妊娠／分娩／産褥期ケアおよび月経障害ケア以外の 10 項目では、50%以上が「好ましい」または「非常に好ましい」と肯定的な意識をもっていた。他国では、男性助産師を好む意見として、女性助産師より男性助産師は優しいとの報告がある [11]。このように、肯定的な意識をもっていた助産ケアにおいては、受け手の女性に拒否されることなく、女性助産師と同等に助産ケアを実施できる可能性が示唆された。

学年別の助産ケアの意識について

学年別の助産ケアの意識おいて、1 年生が他学年に比較して最も否定的な意識をもっていた。一方、2 年生が他学年に比較して最も肯定的な意識をもっており、3～4 年生で再度、否定的な意識が高まっていく傾向があった。1 年生では母性看護学の単位はなく、周産期に関する知識や技術を取得する機会はない。また、調査は入学後 1～2 ヶ月間でデータ収集されているため、医療従事者への関心は高いが看護に関わる全般的な知識や技術もほとんどない状況であった。2 年生では前期で母性看護学概論、後期で母性看護学各論の単位を取得するため、座学による母性看護学に関わる知識を得ている。母性看護学概論では、性に関する講義内容があり、性の多様性や性教育などについて学習する。3 年生では前期で母性看護学演習の単位を取得して、知識に加えて母性看護学に関する技術を得ている。3 年生後期では母性看護学実習の単位において、順番に 2 週間の病院実習があり、産科病棟で褥婦と新生児を受け持ち、看護援助を行っている。多くの学生は外来で妊婦と接し、分娩に立ち合うことも経験している。全ての 4 年生は 1 ～3 年の単位を取得している。これらの教育カリキュラムより、1 年生は周産期に関してほぼ一般的な知識をもった状況での回答であったと考えられる。2 年生では性に関する知識を得ている状況での回答であった。Tian ら（2022）[12] や Gerezgihe ら（2022）[13] が健康教育によって、子宮頸がん検診や医療サービス受診の受入れを高めた結果にあるように、2 年生は性に関する知識を得たことにより、肯定的な意識が高まった可能性が考えられる。しかし、3 年生の技術演習や 4 年生の病棟実習で具体的な助産ケアの実践場面に遭遇したことにより、2 年生の知識のみで抽象的であった助産ケアの内容が具体的にイメージできるようになり、そのイメージが回答に反映されたことが予測される。近年、世界の周産期医療従事者の中で男性助産師の割

合は増えており、周産期医療に貢献している[11]。世界的に男性助産師が活躍している中にあるため、今後、学年別の意識の変化への影響因子を明らかにすることにより、男性のみでなく、LGBTQ にある人が日本で助産ケアを実施することに対する意識について検討する必要があると考える。

助産師資格取得に関する意識について

助産師資格取得に関する意識では、助産ケアと同様に男性およびバイセクシャル男性において否定的な意識が高く、外見が男性である助産師に対して否定的な意識が高いことが示唆された。学年別では、ゲイに対して 4 年生が他の学年と比較して最も否定的な意識が高くなり、唯一、1 年生よりも否定的な意識が高くなった項目である。男性ではなくゲイに特化していることや 4 年生がより否定的な意識をもった点は、病院実習での何らかの予期しなかった体験をしたことやプライベートでの個人的な経験などが影響した可能性があるが、不明な点が多く、その考察は今後の課題の 1 つである。

研究限界

本研究には 3 つの限界がある。第 1 の限界は、本研究では学年別の意識の相違が明らかとなったが、横断研究であるため看護学生の個々の意識の変化を明らかにしていない点である。各学年で対象が異なるので、各学年での相違を変化として捉えるには限界がある。縦断研究で個々の変化を明らかにしていくことが今後の課題である。第 2 の限界は、助産師資格取得に関する意識において、4 年生がゲイに対して最も否定的な意識をもっていた結果の解釈に限界があったことである。この結果に影響する因子を今後明らかにすることが課題である。第 3 の限界は、分析対象が少なかった点であり、個々の影響が大きくなった可能性がある。また、男性の対象が少なく、本研究では統計的検出力の確保と推定の不安定性を避ける目的で分析除外したが、周産期の看護援助に関わる可能性のある男性看護学生の意識調査が今後必要であると考える。

結論

看護学生は、妊娠/分娩/産褥期ケア、月経障害のケアおよび助産師資格取得において、外見が女性であることを好む傾向があった。学年別では 1 年生が他の学年と比較して否定的な意識が高い傾向があった。

利益相反

本研究における利益相反はない。

謝辞

この研究に参加いただいたすべての方に感謝申し上げます。

引用文献

1. Kanai-Pak M. Shogaikoku no kangoshoku no seisa nikansuru jittai nitsuite no kenkyu (A Study

on the Actual Status of Gender Differences among Nursing Professionals in Foreign Countries); 2022 (in Japanese).<https://mhlw-grants.niph.go.jp/system/files/download_pdf/2021/202106024A.pdf>(Accessed July 11, 2025)

2. Japanese Midwives Association. Soritsu 90shunen kinenshi (90th Anniversary Commemorative Publication); 2017(in Japanese).< https://www.midwife.or.jp/user/media/midwife/page/association/ayumi/anniversary_90.pdf >(Accessed July 11, 2025)
3. Dentsu Group Inc. Dentsu Group Conducts LGBTQ+ Survey 2023; 2023(in Japanese). <https://www.group.dentsu.com/jp/news/release/pdf-cms/2023046-1019.pdf> (Accessed June 29, 2025)
4. Japan Lawyers Association for Freedom. Saikosai niyoru seibetsu henko no shujutsu yoken "iken" handan o uke, seiteki mainoritei no genjo ni mukiai, kofuku tsuikyuken o fumaeta shiho to rippo no arikata o motomeru seimei (Supreme Court rules sterilization requirement unconstitutional); 2023 (in Japanese). <https://www.jlaf.jp/04seimei/2023/1027_1647.html> (Accessed June 29, 2025)
5. Sannomiya M, Sasagawa E, Hikita N, Yonezawa K, Haruna M. The Proportions, Regulations, and Training Plans of Male Midwives Worldwide: A Descriptive Study of 77 Countries. Int J Childbirth 2019; 9: 5-18.
6. Japanese Midwives Association. Josanshi no seimei koryo (in Japanese). <https://www.midwife.or.jp/midwife/statement.html> (Accessed June 29, 2025)
7. Suzuki A. Mechanisms of persistence in gender inequality: Perspective of work and family. Japanese Psychological Review 2017; 60: 62–80 (in Japanese).
8. Gilbert TN, Vida NY, ubarick NA, Florence AZ, Hassana AA, Mercy AB, Abigail A. Experiences of women receiving reproductive health services from male midwives: a qualitative study in Bole District, Ghana. BMC Pregnancy Childbirth. 2022;7:30.
9. Wilfred SS, Criscent T, Juliet N. Experiences of mothers receiving male midwives' assistance during childbirth in Kween District, Uganda: a qualitative study. Reprod Health Afr Med J. 2024;28(10):28-38.
10. Courage SM, Maria SM, Lunic BK: Perceptions of postpartum mothers towards the care provided by male student midwives at labour units in Limpopo Province, South Africa. Reprod Health Afr Med J. 2018;22(2):60-67.
11. Ramatu Abdul Hamid Alhassan, Sara Baumann, Abimbola Fapohunda, Janet Awopole Yepakeh Tiah, Timothy Tienbia Laari, Lauren Narbey, Nancy A. Niemczyk, Victoria Bam, Martina Anto-Ocrah: Men as Midwifery Professionals: A Scoping Review. Birth. 2025;52(3):376-392.
12. Yihao Tian, Tao Luo1 and Yuxiao Chen: The Promotional Effect of Health Education on the Medical Service Utilization of Migrants: Evidence From China. Front Public Health. 2022;28:9:818930.

13. Gerezgiher Buruh Abera, Solomon Mekonen Abebe, Abebaw Gebeyehu Worku: Impact of health education intervention on demand of women for cervical cancer screening: a cluster-randomized controlled trial. Research. 2022; Article number: 834.

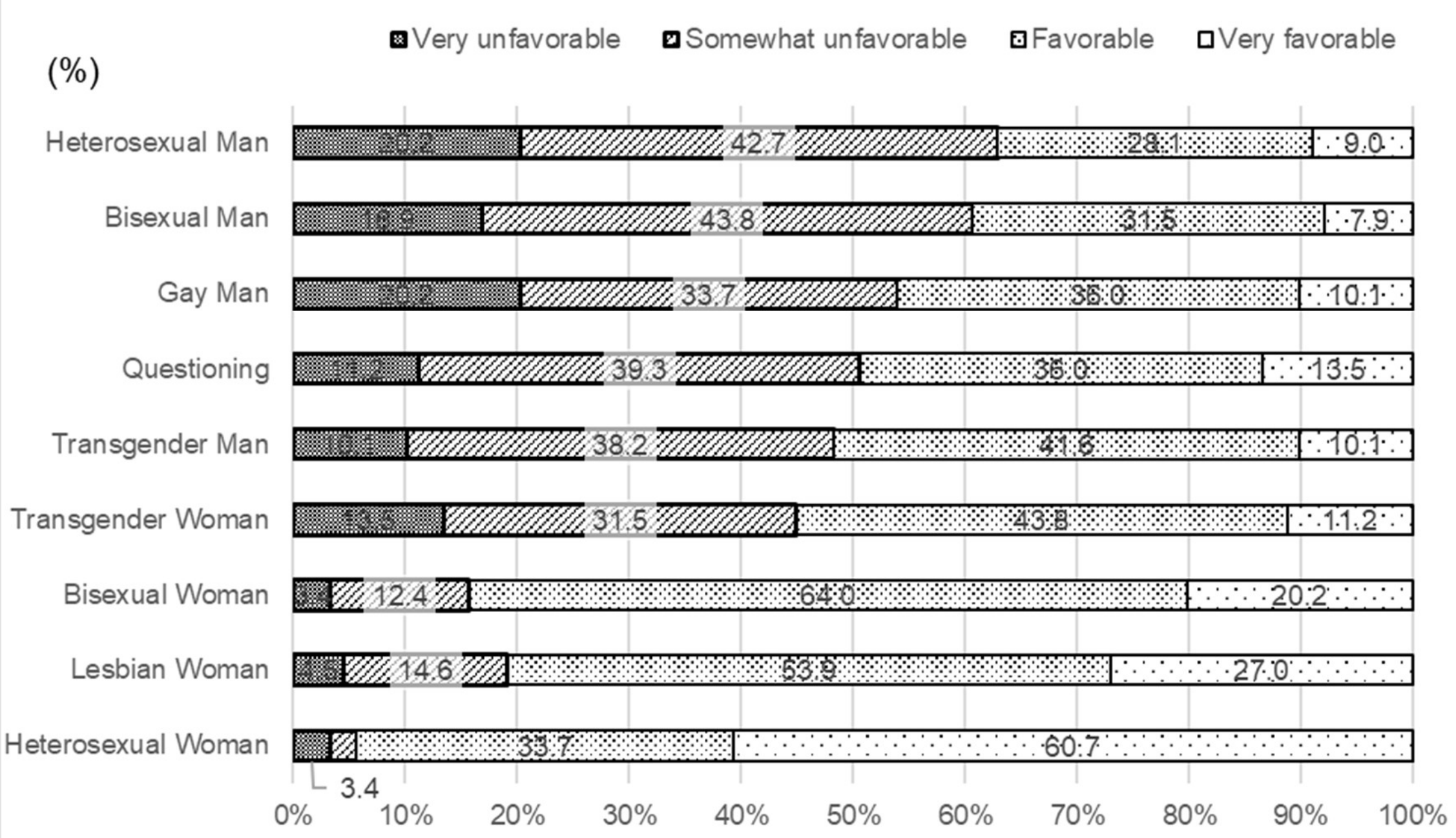

Figure1 Prenatal care

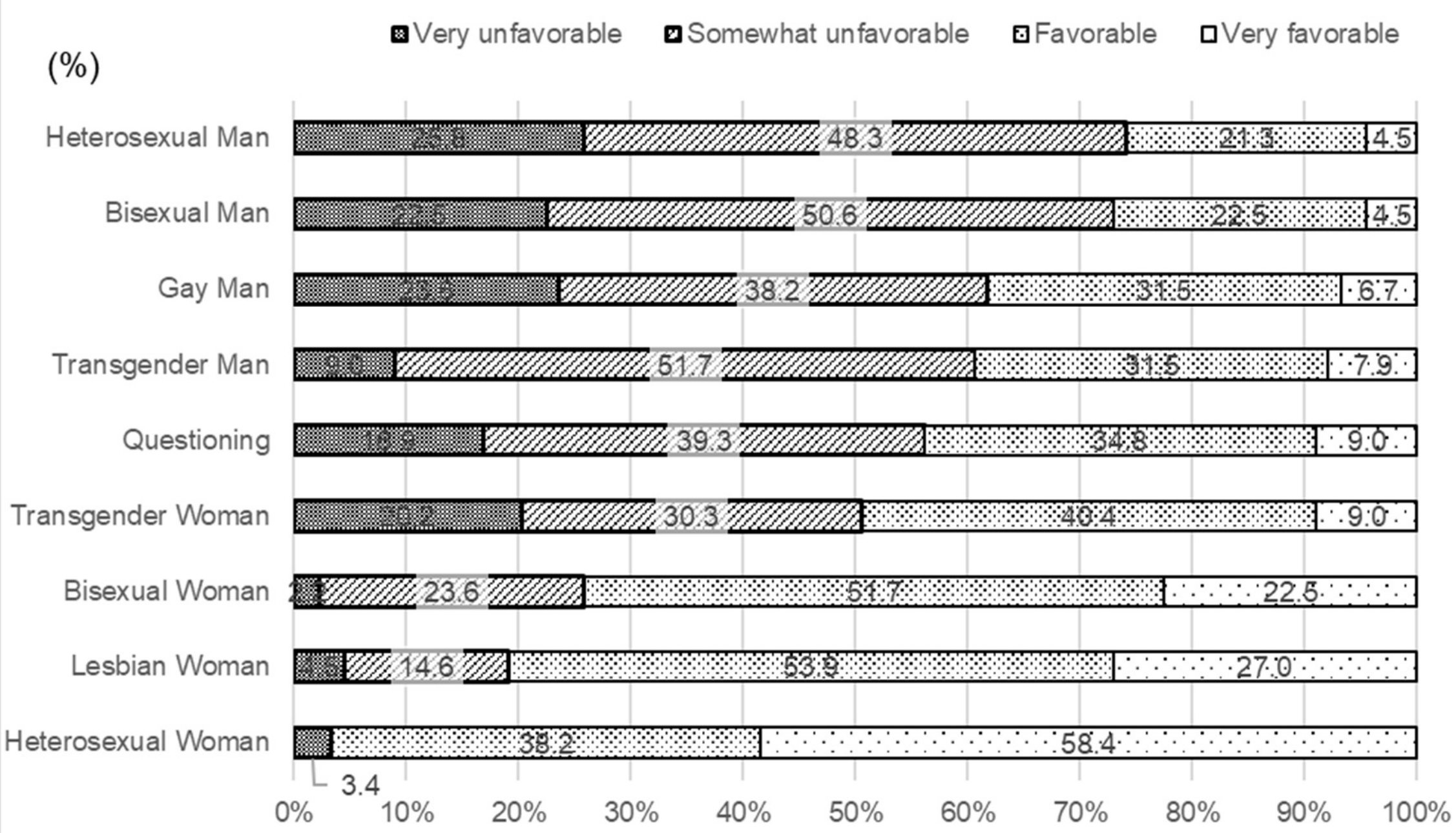

Figure2 Childbirth care

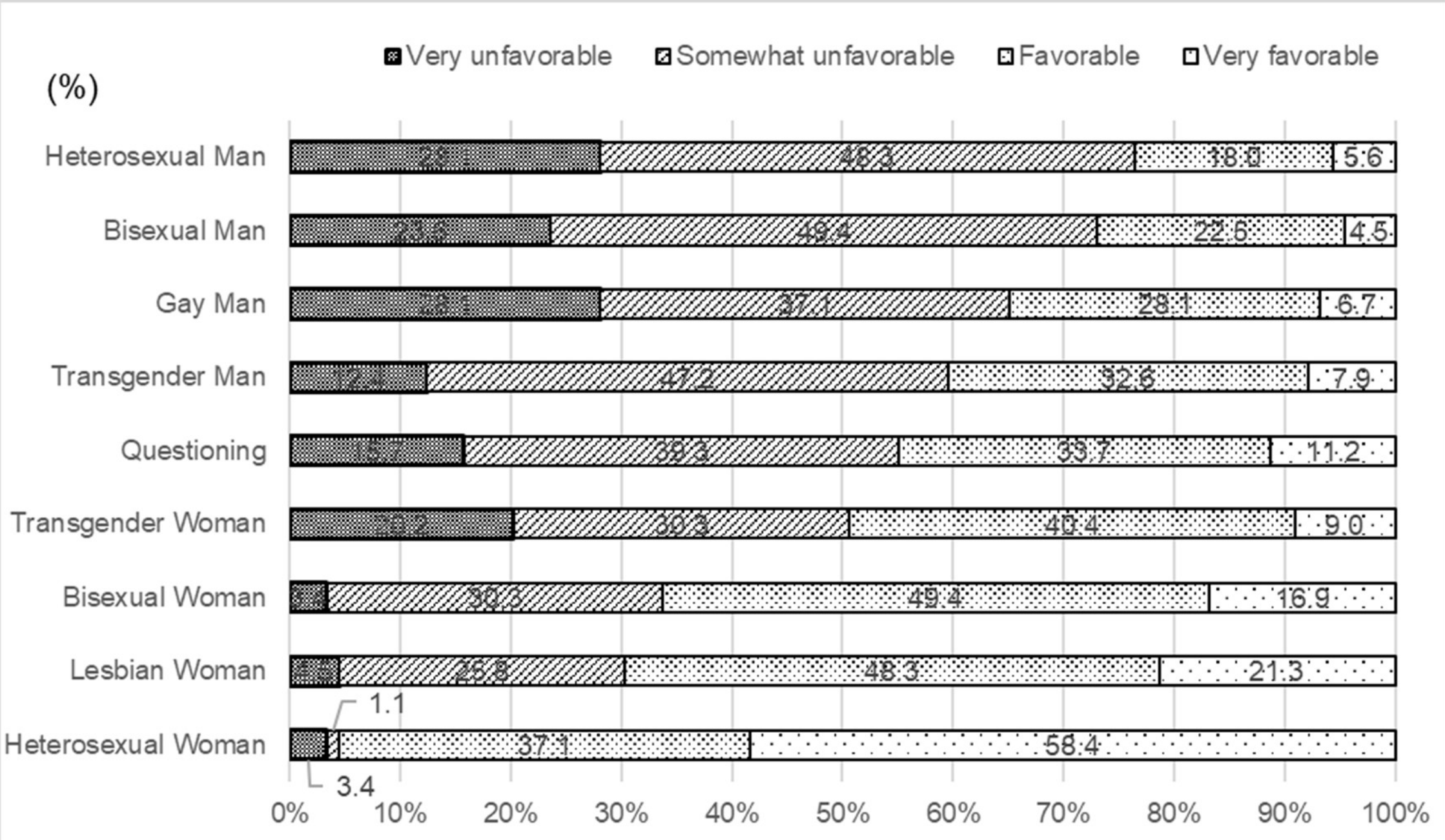

Figure3 Postpartum care

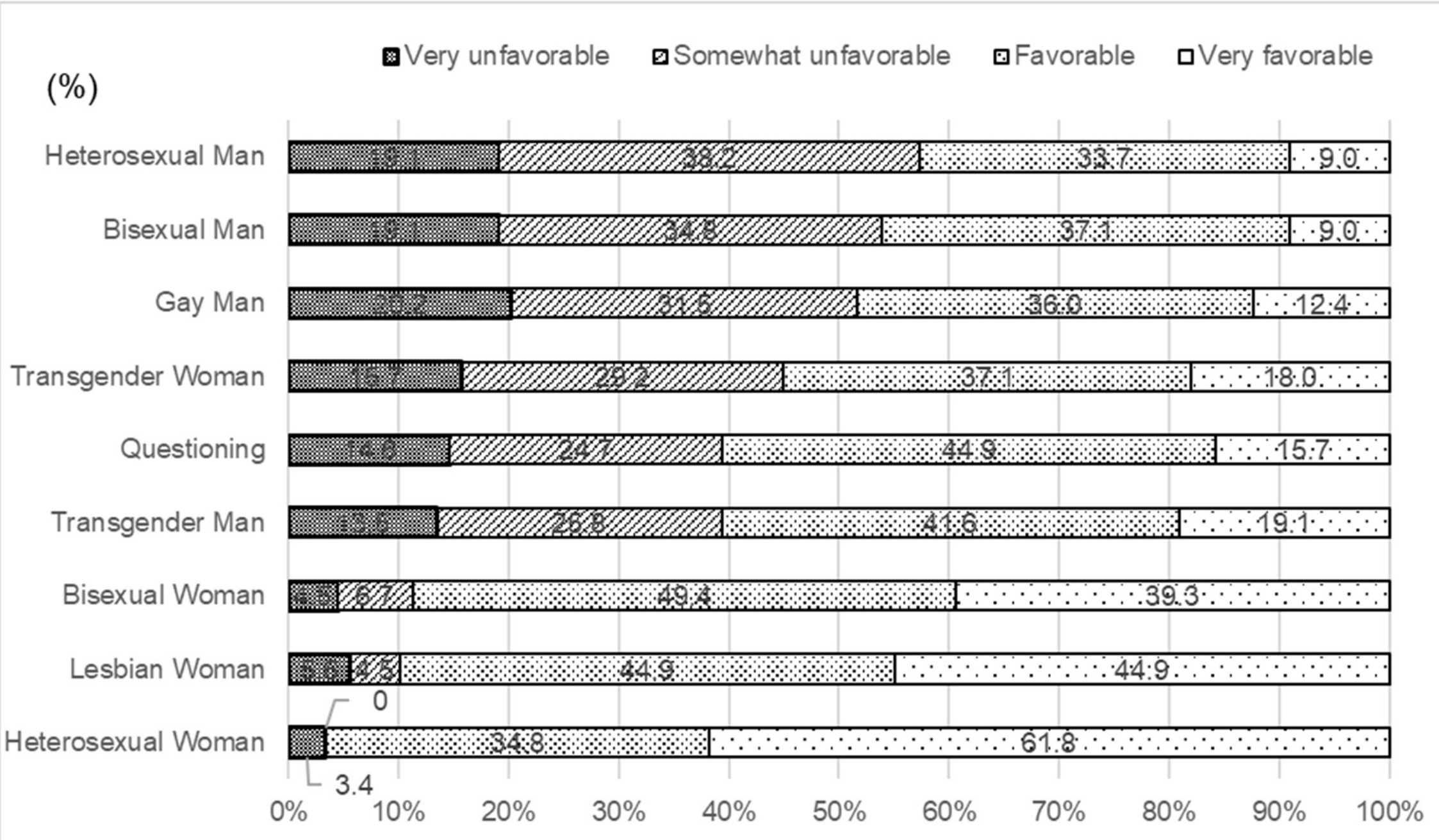

Figure4 Menstrual disorders care

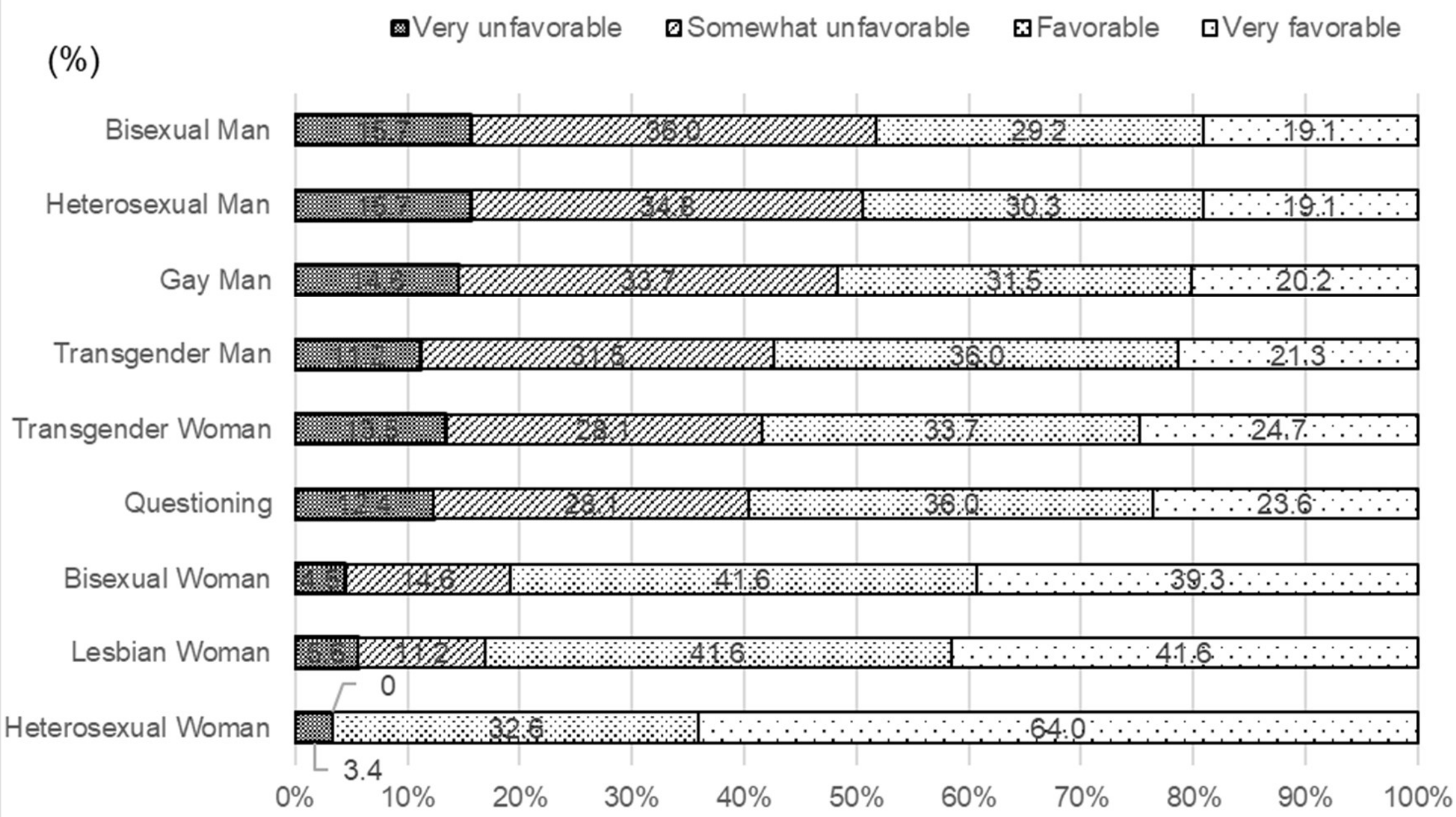

Figure5 Acquisition of Midwifery Qualification

Table1 Midwifery care and Attitudes Toward Obtaining Midwifery Certification by Grade Level

			Grade1(N=20)		Grade2(N=24)		Grade3(N=24)		Grade4(N=21)		χ^2test
			N	%	N	%	N	%	N	%	
Prenatal care	Heterosexual Woman	Negative*	1	5.0%	1	4.2%	1	4.2%	2	9.5%	
		Positive**	19	95.0%	23	95.8%	23	95.8%	19	90.5%	
	Heterosexual Man	Negative	16	80.0%	9	37.5%	15	62.5%	16	76.2%	p<0.05
		Positive	4	20.0%	15	62.5%	9	37.5%	5	23.8%	
	Lesbian Woman	Negative	8	40.0%	2	8.3%	2	8.3%	5	23.8%	p<0.05
		Positive	12	60.0%	22	91.7%	22	91.7%	16	76.2%	
	Gay Man	Negative	14	70.0%	6	25.0%	14	58.3%	14	66.7%	p<0.01
		Positive	6	30.0%	18	75.0%	10	41.7%	7	33.3%	
	Bisexual Woman	Negative	7	35.0%	1	4.2%	2	8.3%	4	19.0%	p<0.05
		Positive	13	65.0%	23	95.8%	22	91.7%	17	81.0%	
	Bisexual Man	Negative	16	80.0%	8	33.3%	15	62.5%	15	71.4%	p<0.01
		Positive	4	20.0%	16	66.7%	9	37.5%	6	28.6%	
	Transgender Woman	Negative	11	55.0%	4	16.7%	14	58.3%	11	52.4%	p<0.05
		Positive	9	45.0%	20	83.3%	10	41.7%	10	47.6%	
	Transgender Man	Negative	13	65.0%	5	20.8%	12	50.0%	13	61.9%	p<0.05
		Positive	7	35.0%	19	79.2%	12	50.0%	8	38.1%	
	Questioning	Negative	16	80.0%	7	29.2%	11	45.8%	11	52.4%	p<0.01
		Positive	4	20.0%	17	70.8%	13	54.2%	10	47.6%	
Childbirth care	Heterosexual Woman	Negative	1	5.0%	0	0.0%	1	4.2%	1	4.8%	
		Positive	19	95.0%	24	100.0%	23	95.8%	20	95.2%	
	Heterosexual Man	Negative	18	90.0%	15	62.5%	18	75.0%	15	71.4%	
		Positive	2	10.0%	9	37.5%	6	25.0%	6	28.6%	
	Lesbian Woman	Negative	10	50.0%	5	20.8%	5	20.8%	7	33.3%	
		Positive	10	50.0%	19	79.2%	19	79.2%	14	66.7%	
	Gay Man	Negative	16	80.0%	10	41.7%	15	62.5%	14	66.7%	
		Positive	4	20.0%	14	58.3%	9	37.5%	7	33.3%	
	Bisexual Woman	Negative	10	50.0%	2	8.3%	4	16.7%	7	33.3%	p<0.01
		Positive	10	50.0%	22	91.7%	20	83.3%	14	66.7%	
	Bisexual Man	Negative	17	85.0%	14	58.3%	18	75.0%	16	76.2%	
		Positive	3	15.0%	10	41.7%	6	25.0%	5	23.8%	
	Transgender Woman	Negative	12	60.0%	9	37.5%	13	54.2%	11	52.4%	
		Positive	8	40.0%	15	62.5%	11	45.8%	10	47.6%	
	Transgender Man	Negative	16	80.0%	12	50.0%	13	54.2%	13	61.9%	
		Positive	4	20.0%	12	50.0%	11	45.8%	8	38.1%	
	Questioning	Negative	17	85.0%	11	45.8%	11	45.8%	11	52.4%	p<0.05
		Positive	3	15.0%	13	54.2%	13	54.2%	10	47.6%	
Postpartum care	Heterosexual Woman	Negative	1	5.0%	1	4.2%	1	4.2%	1	4.8%	
		Positive	19	95.0%	23	95.8%	23	95.8%	20	95.2%	
	Heterosexual Man	Negative	16	80.0%	18	75.0%	19	79.2%	15	71.4%	
		Positive	4	20.0%	6	25.0%	5	20.8%	6	28.6%	
	Lesbian Woman	Negative	11	55.0%	5	20.8%	5	20.8%	6	28.6%	p<0.05
		Positive	9	45.0%	19	79.2%	19	79.2%	15	71.4%	
	Gay Man	Negative	14	70.0%	13	54.2%	17	70.8%	14	66.7%	
		Positive	6	30.0%	11	45.8%	7	29.2%	7	33.3%	
	Bisexual Woman	Negative	12	60.0%	7	29.2%	5	20.8%	6	28.6%	p<0.05
		Positive	8	40.0%	17	70.8%	19	79.2%	15	71.4%	
	Bisexual Man	Negative	16	80.0%	17	70.8%	18	75.0%	14	66.7%	
		Positive	4	20.0%	7	29.2%	6	25.0%	7	33.3%	
	Transgender Woman	Negative	10	50.0%	9	37.5%	14	58.3%	12	57.1%	
		Positive	10	50.0%	15	62.5%	10	41.7%	9	42.9%	
	Transgender Man	Negative	14	70.0%	13	54.2%	14	58.3%	12	57.1%	
		Positive	6	30.0%	11	45.8%	10	41.7%	9	42.9%	
	Questioning	Negative	15	75.0%	10	41.7%	13	54.2%	11	52.4%	
		Positive	5	25.0%	14	58.3%	11	45.8%	10	47.6%	
Menstrual disorders care	Heterosexual Woman	Negative	1	5.0%	0	0.0%	1	4.2%	1	4.8%	
		Positive	19	95.0%	24	100.0%	23	95.8%	20	95.2%	
	Heterosexual Man	Negative	12	60.0%	11	45.8%	12	50.0%	16	76.2%	
		Positive	8	40.0%	13	54.2%	12	50.0%	5	23.8%	
	Lesbian Woman	Negative	5	25.0%	0	0.0%	1	4.2%	3	14.3%	p<0.05
		Positive	15	75.0%	24	100.0%	23	95.8%	18	85.7%	
	Gay Man	Negative	10	50.0%	9	37.5%	12	50.0%	15	71.4%	
		Positive	10	50.0%	15	62.5%	12	50.0%	6	28.6%	
	Bisexual Woman	Negative	5	25.0%	0	0.0%	1	4.2%	4	19.0%	p<0.05
		Positive	15	75.0%	24	100.0%	23	95.8%	17	81.0%	
	Bisexual Man	Negative	12	60.0%	9	37.5%	12	50.0%	15	71.4%	
		Positive	8	40.0%	15	62.5%	12	50.0%	6	28.6%	
	Transgender Woman	Negative	8	40.0%	8	33.3%	11	45.8%	13	61.9%	
		Positive	12	60.0%	16	66.7%	13	54.2%	8	38.1%	
	Transgender Man	Negative	10	50.0%	7	29.2%	8	33.3%	10	47.6%	
		Positive	10	50.0%	17	70.8%	16	66.7%	11	52.4%	
	Questioning	Negative	10	50.0%	8	33.3%	8	33.3%	9	42.9%	
		Positive	10	50.0%	16	66.7%	16	66.7%	12	57.1%	
Attitudes Toward Obtaining Midwifery Certification	Heterosexual Woman	Negative	1	5.0%	0	0.0%	1	4.2%	1	4.8%	
		Positive	19	95.0%	24	100.0%	23	95.8%	20	95.2%	
	Heterosexual Man	Negative	11	55.0%	8	33.3%	11	45.8%	15	71.4%	
		Positive	9	45.0%	16	66.7%	13	54.2%	6	28.6%	
	Lesbian Woman	Negative	6	30.0%	1	4.2%	5	20.8%	3	14.3%	
		Positive	14	70.0%	23	95.8%	19	79.2%	18	85.7%	
	Gay Man	Negative	10	50.0%	7	29.2%	11	45.8%	15	71.4%	p<0.05
		Positive	10	50.0%	17	70.8%	13	54.2%	6	28.6%	
	Bisexual Woman	Negative	6	30.0%	1	4.2%	4	16.7%	6	28.6%	
		Positive	14	70.0%	23	95.8%	20	83.3%	15	71.4%	
	Bisexual Man	Negative	12	60.0%	8	33.3%	11	45.8%	15	71.4%	
		Positive	8	40.0%	16	66.7%	13	54.2%	6	28.6%	
	Transgender Woman	Negative	9	45.0%	7	29.2%	8	33.3%	13	61.9%	
		Positive	11	55.0%	17	70.8%	16	66.7%	8	38.1%	
	Transgender Man	Negative	11	55.0%	6	25.0%	9	37.5%	12	57.1%	
		Positive	9	45.0%	18	75.0%	15	62.5%	9	42.9%	
	Questioning	Negative	11	55.0%	6	25.0%	8	33.3%	11	52.4%	
		Positive	9	45.0%	18	75.0%	16	66.7%	10	47.6%	

*:Negative:includes 'Very unfavorable' or 'Somewhat unfavorable'
**:Positive: incluges 'Very favorable' or ' Favorable'

Supplementary Material 1

(1) Pregnancy care

(2) Childbirth care

(3) Postpartum care

(4) Newborn/infant care

(5) Community maternal and child health care

(6) High-risk, highly advanced medical care

(7) Prenatal diagnosis and genetic counseling

(8) Adolescent care

(9) Middle age care

(10) Family planning care

(11) Women with infertility problems

(12) Sexually transmitted diseases care

(13) Menstrual disorders care

(14) Violence against women Care for violence against women

図 1. 妊娠期のケア

図 2. 分娩期のケア

図 3. 産褥期のケア

図 4. 月経障害のケア

図 5. 助産師資格取得

表 1. 学年別の助産ケアおよび助産師資格取得に関する意識

補足資料 1. 助産師の役割・責務 14 項目
