# Supplementary material for: Attitudes of nursing students toward male and LGBTQ individuals entering the midwifery profession and their role in providing midwifery care
Source: Fujita Med J. 2026 Sep 25;12(4):317–24. doi: 10.20407/fmj.2025-035 (PMC13724197; doi:10.20407/fmj.2025-035)
Supplement: Supplementary file 2 [file fmj-12-317_s2.pdf]

Supplementary Material 1

(1) Pregnancy care
(2) Childbirth care
(3) Postpartum care
(4) Newborn/infant care
(5) Community maternal and child health care
(6) High-risk, highly advanced medical care
(7) Prenatal diagnosis and genetic counseling
(8) Adolescent care
(9) Middle age care
(10) Family planning care
(11) Women with infertility problems
(12) Sexually transmitted diseases care
(13) Menstrual disorders care
(14) Violence against women Care for violence against women
